# Perspectives for the Use of Umbilical Cord Blood in Transplantation and Beyond: Initiatives for an Advanced and Sustainable Public Banking Program in Greece

**DOI:** 10.3390/jcm13041152

**Published:** 2024-02-18

**Authors:** Patra Pateraki, Helen Latsoudis, Anastasia Papadopoulou, Ioanna Gontika, Irene Fragiadaki, Irene Mavroudi, Nikoleta Bizymi, Aristea Batsali, Michail E. Klontzas, Angeliki Xagorari, Efstathios Michalopoulos, Damianos Sotiropoulos, Evangelia Yannaki, Catherine Stavropoulos-Giokas, Helen A. Papadaki

**Affiliations:** 1 Law Directorate of the Health Region of Crete, Ministry of Health, Heraklion, 71500 Heraklion, Greece; eny@hc-crete.gr; 2 Public Cord Blood Bank of Crete, Department of Hematology, University Hospital of Heraklion, 71500 Heraklion, Greece; igontika@pagni.gr (I.G.); fragiada@med.uoc.gr (I.F.); i.mavroudi@uoc.gr (I.M.); nikoletabizymi@yahoo.gr (N.B.); tea_ios@yahoo.gr (A.B.); 3 Institute of Computer Sciences, Foundation for Research and Technology–Hellas (FORTH), 70013 Heraklion, Greece; latsoudi@ics.forth.gr; 4 Gene and Cell Therapy Center, Department of Hematology-HCT Unit, George Papanikolaou Hospital, 57010 Thessaloniki, Greece; papada_1@hotmail.com; 5 Hemopoiesis Research Laboratory, School of Medicine, University of Crete, 71500 Heraklion, Greece; eyannaki@uw.edu; 6 Department of Radiology, School of Medicine, University of Crete, 71500 Heraklion, Greece; miklontzas@gmail.com; 7 Department of Medical Imaging, University Hospital of Heraklion, 71500 Heraklion, Greece; 8 Public Cord Blood Bank, Department of Hematology, George Papanikolaou Hospital, 57010 Thessaloniki, Greece; xagorari.a.gpapanikolaou@n3.syzefxis.gov.gr (A.X.); dsotiro@otenet.gr (D.S.); 9 Hellenic Cord Blood Bank (HCBB), Biomedical Research Foundation Academy of Athens, 11527 Athens, Greece; smichal@bioacademy.gr (E.M.); cstavrop@bioacademy.gr (C.S.-G.)

**Keywords:** umbilical cord blood (UCB), umbilical cord blood banks, hematopoietic stem cell transplantation (HSCT), human leukocyte antigens (HLA), mesenchymal stem cells (MSC), induced pluripotent stem cells (iPSC), plasma rich platelets (PRP)

## Abstract

The umbilical cord blood (UCB) donated in public UCB banks is a source of hematopoietic stem cells (HSC) alternative to bone marrow for allogeneic HSC transplantation (HSCT). However, the high rejection rate of the donated units due to the strict acceptance criteria and the wide application of the haploidentical HSCT have resulted in significant limitation of the use of UCB and difficulties in the economic sustainability of the public UCB banks. There is an ongoing effort within the UCB community to optimize the use of UCB in the field of HSCT and a parallel interest in exploring the use of UCB for applications beyond HSCT i.e., in the fields of cell therapy, regenerative medicine and specialized transfusion medicine. In this report, we describe the mode of operation of the three public UCB banks in Greece as an example of an orchestrated effort to develop a viable UCB banking system by (a) prioritizing the enrichment of the national inventory by high-quality UCB units from populations with rare human leukocyte antigens (HLA), and (b) deploying novel sustainable applications of UCB beyond HSCT, through national and international collaborations. The Greek paradigm of the public UCB network may become an example for countries, particularly with high HLA heterogeneity, with public UCB banks facing sustainability difficulties and adds value to the international efforts aiming to sustainably expand the public UCB banking system.

## 1. Introduction

Umbilical cord blood (UCB) is enriched in hematopoietic stem cells (HSC) and has been used since 1988 as an alternative source to bone marrow (BM) for allogeneic transplantation of patients with hematologic, metabolic diseases and immune deficiencies [1]. The usefulness of the UCB in the field of HSC transplantation (HSCT) has led to the development of UCB banks for the storage of harvested units, following processing and cryopreservation, for future use. Two main types of UCB banks exist, namely, public and private [2]. Public banks are based on the altruistic donation of UCB units and contribute to the coverage of transplantation needs of the general population, on allogeneic basis, according to the human leucocyte antigen (HLA) compatibility and they may also store units for families at high risk for specific diseases. Private banks store UCB units exclusively for autologous or family usage. In some countries, a hybrid bank type combining public and private UCB storage modalities has been developed [3,4,5].

To date, more than 800,000 UCB units are available in the World Marrow Donor Association (WMDA) inventory for allogeneic usage and more than 40,000 transplantations have been performed using UCB for pediatric and adult patients [6]. Recently however, the emergence and successful outcome of HLA haploidentical BM transplantations and their progressive implementation in the practice of transplantation centers worldwide, has resulted in a dramatic global decrease of UCB transplantations [7]. The consequent low shipping rate of the UCB has increased the economic vulnerability of the public banks which were already facing financial difficulties due to the high running costs and the high discard rate of the donated UCB units. Specifically, given that the successful outcome of the UCB transplantation is largely dependent on the qualitative and quantitative characteristics of the HSC in the units, strict criteria have been introduced by the international Accreditation Bodies and the ethnic legislations for the collection, banking, and release of the UCB for transplantation purposes [8,9]. For example, an adequate volume of the donated UCB units and a relatively high threshold of total nucleated cells (TNC) and CD34^+^ cells, corresponding to the HSC content of the units, are required to start the processing procedure. The recommended prerequisites result in a rejection rate of approximately 90% of the donated units that apparently affects the financial sustainability of the public UCB banks and the altruistic enthusiasm for donation [2].

Despite the above difficulties, UCB remains undoubtedly an important component of the alternative donor pool together with the family of HLA-haploidentical and the unrelated HLA-mismatched donors. The advantage of the rapid availability of UCB for urgent situations or for ethnic groups that are under-represented in international registries, coupled with the comparable or even superior data of CB versus haploidentical transplantations in pediatric populations and in patients with non-remission acute myeloid leukemia, highlight the usefulness of the UCB and indicate that, overall, the alternative donor source selection needs an individualized approach that should take into consideration patient-, disease-, and transplantation-related factors [10,11,12,13].

Undoubtedly, the high-quality UCB units registered today in the international inventories offer life-saving opportunities for patients in need of allogeneic HSCT; therefore, development of sustainability strategies for the long-term survival of the public UCB banks are of particular significance. Thus, there is an ongoing effort within the UCB community for the harmonization and optimization of the collection, processing, storage and typing of the UCB prioritizing the high TNC and CD34^+^ cell content and the HLA diversity of the units [14]. Such an approach is expected to increase the accessibility and trust by the transplant centers, and also to improve the efficiency and cost of the UCB banking. During this process, a parallel interest has been developed in exploring the use of UCB as a source for applications beyond the HSCT i.e., in the fields of (a) cell therapy following cell expansion, engineering or reprogramming, (b) regenerative medicine, (c) specialized transfusion medicine, (d) research for identification of novel cellular components and cell derivatives [14,15,16,17]. In all these applications, the starting material might even be units unsuitable for HSCT, thus contributing to the sustainability of the banks and the recycling of the biologic material [18].

Initiatives supporting this new concept of banking that aims to increase the therapeutic value of the UCB and minimize the financial deficit of the public system, have been emerged as individual or collaborative efforts of public UCB banks across Europe [18,19,20,21,22,23]. The main objective of the current report is to describe the mode of operation of the three public UCB banks in Greece (located in the University Hospital of Heraklion, Crete; in the Biomedical Research Foundation of Academy of Athens; in George Papanikolaou General Hospital of Thessaloniki) as an example of an orchestrated effort to develop and sustain a viable UCB banking system. We describe the joint policy (a) to prioritize the enrichment of the national inventory by high-quality units particularly from populations with rare HLA across the country, and (b) to develop novel sustainable applications of UCB beyond HSCT, in the fields of cellular therapies and regenerative and transfusion medicine, through national and international collaborations that implicate bank specialists, researchers and transplant physicians. The Greek paradigm of the public UCB network may become an example for countries, particularly with high HLA heterogeneity, with public UCB banks facing sustainability difficulties.

## 2. UCB Banking for Allogeneic HSCT: The Greek Policies for Efficacy Optimization Based on the Population HLA Profiling

The three public UCB banks in Greece operate under the Hellenic Transplant Organization (HTO) and the Ministry of Health. According to the national legislation the collection, processing, storage and characterization of the UCB units should follow the NetCord-Foundation for Accreditation of Cell Therapies (NetCord-FACT) standards to be listed in the WMDA international inventory [9]. The Greek UCB banks cooperate with European establishments to share good practices through their involvement in European projects aiming to homogenize and improve the preparation processes for blood, tissues and cells such as the GAPP (facilitating the authorization of preparation process; https://www.gapp-ja.eu/ (accessed on 15 February 2024) ) and EGALITE (European group for accreditation and liaison of blood-tissues and cells establishments; https://www.egalite-europe.eu/ (accessed on 15 February 2024)) Actions. At the national level, the UCB banks have developed common policies to promote the altruistic donation of UCB and also strive for the improvement of quality of the donating units through close collaboration with the collection teams, obstetricians, labor and delivery staff.

Currently, a specific approach is being developed at the national level aiming to optimize the efficiency of UCB banking based on the HLA profiling of the Greek population. Specifically, it is known that HLA harbors unusually complex sequence variations that are specific to individual ancestral populations; thus, the distribution of HLA alleles is highly variable across different ethnic groups [24]. The high population-specific genetic diversity of HLA, combined with the complexity of its molecular variation and the limitations of traditional HLA typing, confines the effectiveness of BM donor registries and public UCB banks [24,25]. Such a difficulty is particularly evident in countries like Greece that include ethnic minorities and are also characterized by distinct population structures related to the geographic/genetic isolation, inbreeding or common founders [26].

**Figure 1:**
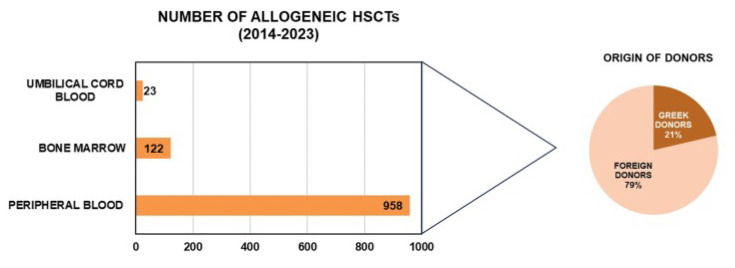
Number and origin of allogeneic HSCTs performed in Greece from unrelated donors during the period 2014–2023. The bar chart shows the hematopoietic stem cell source i.e., peripheral blood, bone marrow, umbilical cord blood. The pie chart shows the origin of the donors. (Abbreviations: HSCT, hematopoietic stem cell transplantation).

According to the data provided by the HTO, over the past 10 years 1103 allogeneic HSCTs have been performed in Greece from unrelated donors. The cell source was peripheral blood in 958, BM in 122 and UCB in 23 of them, whereas only 236 originated from Greek donors (Figure 1). The lack of self-sufficiency despite the increasing number of BM and UCB donors reflects the HLA heterogeneity of the Greek population and highlights the importance of increasing the diversity rather that the size of the national repository [27].

The island of Crete in Greece is an elegant example of a high degree intra-and inter-population HLA diversity probably associated with the mountainous geography and the wealth of historical events that have influenced the genetic history and cultural characteristics of the population [27,28,29,30,31,32]. Such diversity was recently shown in a study comparing the Cretan population with donors from various populations registered in one of the most representative European unrelated donor registries, namely the Deutsche Knochenmarkspenderdatei (DKMS) [27]. Specifically, 15 unique alleles have been detected in Crete that were absent in the DKMS cohort and 8 novel alleles have been reported that contributed to the Cretan HLA variability [27]. Further stratification of the Cretan cohort by the four Prefectures of origin unraveled significant differences in HLA haplotype distribution suggesting a potential role of the demographic history on HLA heterogeneity of specific regions of Crete [28].

Based on these findings and in order to substantiate the importance of geographically and/or genetically isolated population clusters in regional UCB banking and donor selection, the network of the three public UCB banks of Greece in collaboration with the HTO currently evaluate and map the immunogenetic profile of representative cohorts of Greek UCB and BM donor registries (Hellenic Foundation for Research and Innovation grant #44201/2022–2024). HLA alleles and haplotypes frequencies are calculated for the 13 Regions of Greece and subsequent values, which are stratified by place of donor’s origin, are compared not only to each other but also to different populations from established international registries. Such comparative analyses will unravel whether the combination of inter- and intra-population differences in the distribution of HLA haplotypes may unequivocally influence the donor search not only across countries, but also within the country of interest.

In practice, such an approach will develop a model to increase the efficacy of the public UCB banks and BM registries based on the population HLA diversity. Moreover, this will be an overarching reference dataset of high-resolution HLA profiles of the Greek population contributing in the effective donor search and complex disease etiology or susceptibility.

## 3. Novel Applications of UCB beyond HSCT: Insights from the Greek Experience

The criteria for UCB selection for allogeneic HSCT in the Greek UCB banks are those proposed by the NetCord-FACT (Table 1) [9].

UCB units that do not fulfil the criteria described in Table 1 can be used for research purposes as described the below.

### 3.1. UCB-Derived, off-the-Shelf Adoptive Immunotherapy

Adoptive immunotherapy using either genetically- or non-genetically-modified T-cells derived from various sources, has emerged in the recent years as a powerful therapeutic alternative for patients with hematologic malignancies having limited therapeutic options [29,30,31]. In immunocompromised individuals, opportunistic infections can also be effectively managed using adoptive immunotherapy with pathogen-specific T-cells. These specific T cells are expanded ex vivo from the memory T-cell compartment of donors who have previously been exposed to the targeted pathogen, and infused to the patient upon reactivation of the infection [30,32]. The feasibility of establishing T-cell immunotherapy banks with “off-the-shelf” products which can serve as an “on demand” treatment for patients sharing a minimum HLA matching with the donors, has been shown for both hematologic malignancies and opportunistic infections, mainly with T-cell products originating from adult subjects [33,34].

**Figure 2:**
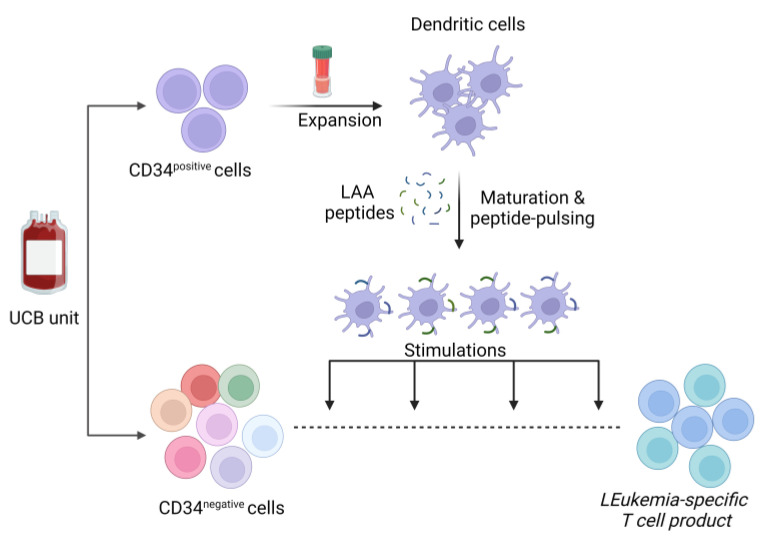
Schematic overview of generating Leukemia-specific T cell from UCB. Immunomagnetically selected CD34^+^ cells from UCB, are expanded in G-rex bioreactors and then differentiated into dendritic cells. These dendritic cells, once matured, are pulsed with overlapping peptides spanning various leukemia-associated antigens (LAAs), enabling them to subsequently serve as antigen-presenting cells for the stimulation of CD34^−^, T cell fraction from UCB. Following four rounds of stimulation, the protocol efficiently yields leukemia-specific T cells. (Abbreviation: UCB, umbilical cord blood).

UCB as a rich source of CD34^+^ and naïve T-cells can be used to increase the scalability of professional antigen-presenting cell production for efficient T-cell priming and ensure a diverse and potent immune response against a spectrum of antigens. We have recently introduced a model of “circular economy” by repurposing the non-transplantable UCB units, to obtain dendritic cell (DC) doses at a ×10^9^ scale and subsequently facilitate the yield of clinically relevant quantities of third-party, bivalent leukemia-specific T-cells (Leuk-STs), targeting Wilms tumor 1 (WT1) and preferentially expressed antigen in melanoma (PRAME) [35]. These non-genetically engineered T-cells, targeting two of the most common transcription molecules overexpressed in leukemias, presented, after 4 stimulations with overlapping peptides spanning the whole WT1 and PRAME antigens, a memory phenotype with high specificity and cytotoxicity against both leukemia-associated antigens (LAA) in vitro. By targeting a plethora of antigen epitopes and expressing central-, effector-memory and TEMRA-markers, Leuk-STs provide the benefits of both, minimization of the tumor escape risk as well as enhanced antitumor responses and in vivo persistence, respectively. Furthermore, ex vivo expanded antigen-specific T-cells from peripheral blood against LAA or pathogens have demonstrated a favorable safety profile in numerous clinical trials up to date where the risk of graft-versus-host disease (GvHD) or cytokine release syndrome (CRS) was minimal, even in a third-party setting [33,34,35,36,37]. Given the unique properties of UCB, allowing for low alloreactivity and no stringent HLA matching over adult blood [38], UCB-derived, antigen-specific T cells could be considered immunologically safe (Figure 2).

Bollard’s group has demonstrated the safety and feasibility of using the 20% fraction of UCB units for expanding UCB-virus-specific T-cells (UCB-VSTs). Administered post UCB transplantation, UCB-VSTs displayed prolonged persistence, restoring antiviral immunity and effectively preventing or treating viral infections against active cytomegalovirus (CMV), Epstein Barr virus (EBV), or adenovirus infections, especially in high-risk patients [39]. By adapting our previously developed protocol of generating Leuk-STs from “recycled” UCB units, we have also generated a compact, “all-in-one”, T-cell product of UCB origin, called “LEVIS” (LEukemia-VIrus-specific T-cells), simultaneously targeting four viruses and two common LAAs [40]. This strategy of “recycling” the disqualified UCB units to serve as an unlimited source for scalable production of antigen-specific T cells, has the potential to broaden the target repertoire and the indications, improve the cost-effectiveness of the products and provide universally accessible immunotherapies.

Other groups have expanded the applications of UCB in cancer immunotherapy by generating UCB-derived natural killer (NK) cells which have been successfully used against hematologic malignancies [41,42] and shown preliminary potential against solid tumors [43,44]. UCB has also been used as a source of T-cells and more recently, NK cells for genetic engineering with chimeric antigen receptors (CARs) [45]. Opting for CAR-NK cells over CAR-T-cells offers several advantages, such as the considerably reduced risk of CRS and neurotoxicity, the dual tumor cell recognition via CAR-dependent and CAR-independent pathways, and the potential for allogeneic application without inducing GvHD or host-versus-graft rejection [46]. However, the durability of tumor responses with CAR-NK cells remains unclear [47]. Genetically-engineered UCB-derived T-cells (CD19-CAR-T) or UCB-derived NK cells (CD19-CAR-NK) have shown cytolytic activity in vitro and in vivo, in models of B-lineage acute lymphoblastic leukemia (B-ALL) or in patients with relapsed or refractory B-cell hematologic malignancies [48,49].

Other UCB-derived immune cell populations, such as the myeloid derived suppressor cells (MDSCs), are also under investigation for their potential clinical application in immunotherapy. MDSCs are immature myeloid cells with immunosuppressive properties against a variety of cell types such as T-cells, NK cells, DCs and macrophages [50]. They are produced in the BM and they are subdivided into two major subgroups, namely the monocytic MDSCs (M-MDSCs) and polymorphonuclear MDSCs (PMN-MDSCs). Studies have shown higher frequency of MDSCs in the UCB of neonates compared to the peripheral blood of adults or children and MDSC numbers decrease dramatically during the first month of life [51]. A potential role for UCB MDSCs in maintaining maternal–fetal tolerance has been postulated. UCB-derived MDSCs have gained particular interest for the treatment of autoimmune and chronic inflammatory diseases and GvHD [52,53]. Specifically, a number of studies using mouse models of GvHD have shown that UCB-derived MDSCs can inhibit GvHD toxicity by suppresing T-cell mediated allogeneic reaction and enhancing T-regulatory cell activity [53]. The isolation, characterization, storage and expansion modalities of UCB derived MDSCs for their adoptive transfer potential is an active field of research in the Greek public UCB banks [54].

It is obvious that collaborative initiatives between the public UCB banks and research institutions have the potential to shape a new therapeutic landscape, offering transformative outcomes for patients requiring immunotherapies and treatments with advanced therapy medicinal products.

### 3.2. UCB and Cord-Tissue Derived Mesechymal Stem/Stromal Cells

**Figure 3:**
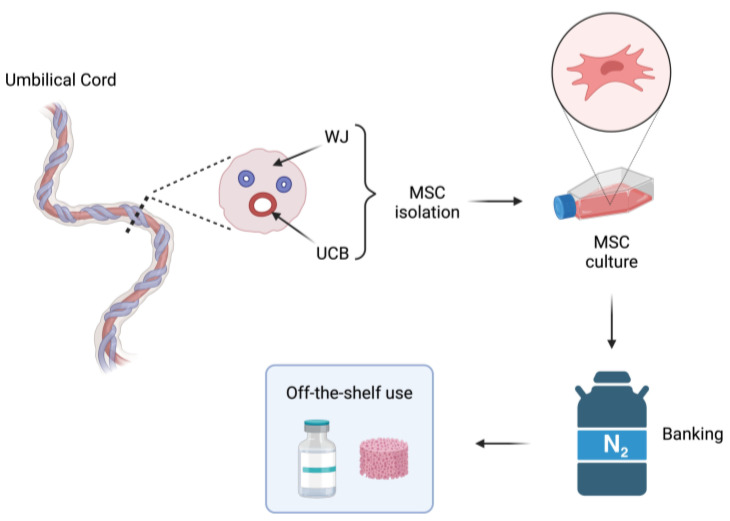
Schematic overview of the isolation and storage of umbilical cord blood (UCB) and Wharton’s jelly (WJ) mesenchymal stem cells (MSCs). Cells are isolated from different umbilical cord compartments, expanded in culture, and banked for the off-the-shelf use.

Mesenchymal stem or stromal cells (MSCs), according to the criteria set by the International Society for Cellular Therapy (ISCT), can be isolated from numerous fetal and adult tissues and represent attractive tools for regenerative medicine due to their multilineage differentiation potential across germ layers and their immunosuppressive properties [55,56]. UCB and Wharton’s Jelly (WJ) have been widely used as fetal MSC sources. Compared to adult tissues such as BM and adipose tissue, UCB and WJ MSCs display significantly enhanced proliferation capacity and can be cultured for an extended number of passages before entering a state of senescence [57,58,59]. However, the extraction efficiency of UCB MSCs is considerably lower, ranging between 10–60% among studies, compared to WJ MSCs that exhibit an isolation efficiency of 100% [60,61]. Thus, the Greek UCB banks have particularly focused on the study of WJ MSC properties for expansion, differentiation and storage (Figure 3).

Studies from the Greek UCB banks have shown that compared to BM MSCs, WJ MSCs display higher proliferative potential due to differential expression of genes related to cell cycle but inferior differentiation capacity toward osteocytes and adipocytes due to altered expression of molecules related to WNT signaling and inferior hematopoiesis-supporting potential associated with reduced production of Stromal cell-Derived Factor-1α [62]. Collaborations with material scientists, however, have shown that suitable scaffolds and culture technologies with appropriate growth factors may induce the osteogenic potential of WJ MSCs for future clinical applications [63]. Collaborative studies have also shown that WJ MSCs display an excellent response on biomaterials generated for myocardium tissue engineering, paving the way for WJ MSC-based myocardium regeneration [64].

Based on the current interest in the use of WJ MSCs for clinical applications, scientists from the Greek UCB banks have developed a vitrification method for the long-term storage of WJ tissue that better supports the extracellular matrix structure and maintains the MSC survival, proliferation and differentiation compared to conventional cryopreservation methods [65]. Therefore, the WJ tissues can efficiently be preserved over a long period at low temperatures with the established vitrification method facilitating the generation of a repository of WJ tissues that would increase the MSC availability for clinical applications.

Overall, the off-the-shelf use of UCB and particularly WJ derived MSCs has been proposed as an excellent atraumatic method for tissue engineering, immunosuppressive and regenerative applications. Compared to MSCs isolated from adult tissues, UCB or WJ derived MSCs have shown consistency in differentiation and proliferation capacity at early, medium and late passages and this consistency is also observed among different donors [66]. The importance of future applications of WJ MSCs has been recognized by the International Standards Organisation’s (ISO) technical committee (TC) which has collaborated with the ISCT to publish standards for MSC isolation, growth and banking [67]. Such standards have been developed to facilitate the authorization of MSC-based products for cellular therapies and future clinical studies. Furthermore, banking of cord tissue has been emerged as an important strategy in parallel to UCB storage [68]. Notably, given that the cord tissue is not a cellular suspension, additional regulatory issues should be addressed at the European and national level.

### 3.3. UCB-Derived Induced Pluripotent Stem Cells from Homozygous Units

The induced pluripotent stem cells (iPSCs) are generated from human somatic cells by reprogramming technology to regain an embryonic ‘stemness’, which not only enables their differentiation into any cell type but also bypasses any ethical considerations since they no longer derive from human embryos or oocytes [69]. The UCB banks are an excellent source of starting material for the production of iPSCs because the UCB is rich in stem cells with fewer genetic or epigenetic changes compared to adult sources, and the units are HLA-typed and stored under clinical-grade and good manufacturing practice (GMP)-standards [23,70].

There is a current interest in the scientific community for the development of super donor cell lines from UCB units homozygous for the top common HLA haplotypes that could provide close immunological match to the vast majority of the population under study [23,70,71,72,73]. Several repositories (haplobanks) have already been created across the world that develop and provide the respective population-specific haplolines to clinical trials whereas a Cooperation in Science and Technology (COST) Action (HAPLO-iPS, https://www.cost.eu/actions/CA21151/ (accessed on 15 February 2024)) has been launched to promote further this concept across Europe [23,74,75,76].

**Figure 4:**
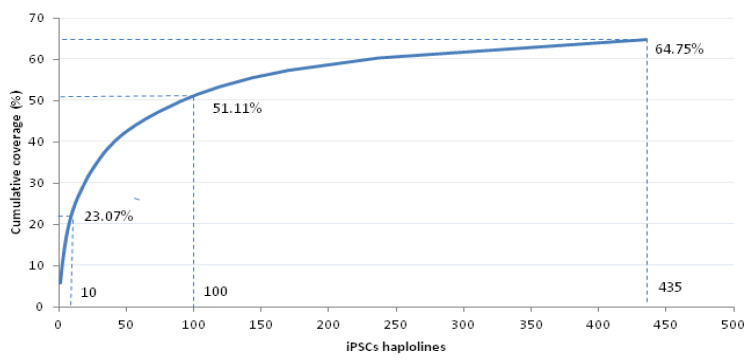
Estimated numbers of iPSC lines homozygous for HLA-A, HLA-B, and HLA-DRB1 (haplolines) to cover the Greek population (cumulative haplotype matching coverage, %).

Preliminary results based on 2869 UCB units from the three Greek public UCB banks participating in the HAPLO-iPS COST Action, have shown 3 homozygous for the 3-HLA loci haplotype A*02:01~B*18:01~DRB1*11:04 on the basis of the suggested coverage matching parameters related to the number of homozygous CB units (i.e., ≥2) and to the haplotype frequency (i.e., ≥1%) [23]. It is estimated that these homozygous UCB units can cover the transplantation needs of 5.68% of the Greek population. It has also been shown that the top 10 and 100 common estimated haplolines can cover 23.07% and 51.11% of the Greek population, respectively, highlighting the diversity of the Greek population (Figure 4).

Even though these results are preliminary, they signify the importance of a broad collaboration in establishing iPSCs repositories from individuals’ homozygous HLA haplotypes to cover the regional HLA diversity. Creating a European or even worldwide Registry of haplobanks could overcome the barrier of inter-and intra-population HLA diversity that significantly affects the search of matched unrelated donors across or within countries. Overcoming allogeneic barriers in regenerative medicine may result in effective standardized “off-the-shelf” cellular products for regenerative therapeutics.

### 3.4. UCB-Derived Plasma Rich Platelet and Related Components

The UCB-derived platelet-rich-plasma (PRP), defined as the fraction of UCB plasma with platelet concentration above baseline following appropriate preparation, used as platelet lysate (PL) or platelet gel upon activation (PG), has been shown to display tissue healing and regenerative properties due to its high content in growth and trophic factors, angiogenic and immunomodulatory cytokines [77]. A number of clinical studies have been performed using allogeneic UCB PRP derivatives and have shown their beneficial effect in a variety of conditions such as dystrophic epidermolysis bullosa, oral mucositis after chemotherapy, chronic ulcers associated with diabetic foot, and perianal fistula [78,79,80,81,82,83]. Furthermore, UCB derived PL has been used in the treatment of severe ocular surface lesions unresponsive to conventional treatments [84].

In view of the interest in exploring the use of UCB PRP derivatives for tissue repair and regeneration but also the recycling potential of the waste UCB units, scientists from the Greek UCB banks have evaluated the qualitative properties of PL obtained from small volume, non-suitable for HSCT UCB units, in terms of growth factor content, wound healing and angiogenesis properties in vitro [85,86]. It was found that despite the lower platelet numbers of small volume UCB units compared to intermediate and higher volume units, the level of angiogenesis promoting cytokines such as platelet derived growth factor-BB (PDGF-BB), transforming growth factor-beta 1 (TGF-β1), fibroblast growth factor (FGF), vascular endothelial growth factor-A (VEGF-A), hepatocyte growth factor (HGF) as well as the immunomodulatory biomolecules indoleamine 2,3-dioxygenase (IDO) and prostaglandin E2 (PGE2) were high enough to promote normal regenerative properties in vitro. These data encourage the use of small volume UCB units for the preparation of PRP derivatives for various clinical applications in regenerative medicine.

Based on the above and other results supporting the regenerative properties of the UCB products, the Greek public UCB banks have participated in an international network of public UCB banks coordinated by the Banc de Sang i Teixits (BST) Barcelona, Spain aiming to promote the recycling of the waste UCB units using homogenized procedures [22]. The initiative has resulted in the generation of protocols for the fractionation of the clinically inappropriate UCB units into (a) UCB PRP for the use in skin (CB-PG) and corneal (eye drops) ulcers, (b) UCB platelet poor plasma (PPP) for the preparation of eye drops for the use in dry eye diseases, and (c) leukoreduced red blood cells (LR-RBC) for possible transfusion of neonates following irradiation. Further to the development of homogenized protocols, the initiative also included multicenter exercise to validate the aforementioned procedures, thus opening the way for large clinical studies to support the multicomponent efficacy of the UCB units in the fields of regenerative and transfusion medicine. Interestingly, based on the available scientific evidence, the Decree of Italian Ministry of Health has allowed the use of UCB-derived PRP, PG, and eye drops not only for in-hospital applications but also for industrial manufacturing opening the way for the collaboration between CB banks and industrial stakeholders that will improve the banks’ economic sustainability [18].

Using validated protocols, the public UCB bank of Crete in collaboration with the Vascular Surgery Department of the University Hospital of Heraklion have substituted the standard of care treatment of diabetic patients with peripheral arterial disease and critical limb ischemia using autologous PRP [87] with UCB-derived PRP from waste UCB units. The current unpublished results from the ongoing study show that this approach is more efficient and cost effective.

### 3.5. UCB-Derived Microparticles

Microparticles (MPs) are subsets of extracellular vesicles 100 to 1000 nm^2^ in size that are released from cells during apoptosis or activation processes [88]. These particles emerge through plasma membrane budding, facilitated by cytoskeletal rearrangements due to elevated intracellular calcium levels. Phosphatidylserine is exposed on the MP surface enabling their identification by flow cytometry using annexin-V. MPs carry also specific cell surface markers originating from their parental cells, thus making it feasible for them to be further recognized by flow cytometry. Remarkably, MPs derived from platelet and endothelial cells have been observed in the peripheral blood of transplanted patients whereas in allogeneic HSCT mouse models, MPs have been shown to enhance engraftment [89,90]. MPs derived from embryonic stem cells possess the capacity to reprogram hematopoietic progenitors and facilitate their differentiation by transporting molecules such as mRNAs, proteins, and miRNAs [91,92,93].

A number of studies have shown the presence of C34^+^ MPs enriched in miRNAs in UCB [94]. Specifically, distinct expression patterns have been identified for miRNA-106, miRNA-517c, miRNA-518a, miRNA-519d and miRNA-520h known to be implicated in the differentiation, survival and engraftment processes of hematopoietic cells [94,95]. Under the prism of identification of novel application of UCB, scientists in the Greek UCB banks are currently investigating the distinct signature characteristics of MPs derived from UCB CD34^+^ cells and their potential use in the induction of apoptosis of leukemic cells [96]. Preliminary data have shown that UCB CD34^+^ MPs display an apoptosis promoting effect on the acute promyelocytic leukemia cell line HL60 and the lymphoblastic leukemia cell line MOLT4. These data inspire further the interest for exploring the mechanism underlying the anti-leukemic effect of UCB derived CD34^+^ MPs and their potential use in the clinic.

Representative studies from the Greek public UCB banks aiming to promote novel fields of research are summarized in Table 2.

## 4. Ethical and Legal Issues

In the current document, we have described a number of procedures by which altruistically donated UCB units, mostly unsuitable for HSCT, can be used for research and novel clinical purposes in fields within and beyond transplantation. Given that more than 90% of donated units are not finally appropriate for storage [2], the emerging field of alternative use of the waste units is of particular importance. In Greece, similar to most European countries, a definite regulatory framework has not been set for the use of UCB units in fields other than HSCT. Specifically, according to the Greek legislation, the UCB collection is based on the written consent of the parents for the donation of the UCB for HSCT purposes (article 48 of the Greek Law 3984/2011). The parents are informed by the staff of the UCB bank for the possibility of unsuitability of their donation for HSCT and they are given the possibility to consent for the use of their UCB unit for research. However, there are legal and ethical challenges that need to be seriously considered and improved.

The main concerns are related to the informed consent of the parents for the development of research products from their altruistic donation. It is necessary to provide the appropriate information regarding all possible options of disposition of the donated UCB units, including donation toward biomedical research, giving in parallel the option to the parents to refuse the use of the units in purposes other than transplantation. Information should be given on an individual basis, in a simple and understandable way, adapted to the educational level of the parents, and should describe the content of the research program(s). It must be in the form of a written document and must include the purpose of the research, the reasons why it is desirable to participate, the methodological focus of the research and the benefits expected from it. The research projects should be approved by the Institutional Scientific Council and the competent Ethics Committee. Particularly for the generation, banking and use of UCB-derived homozygous iPSCs, donor re-contact, clear information and reconsent is necessary since the original UCB donation had been purposed for HSCT.

Personal data should be also protected at all stages of UCB or UCB-derivatives processing including the collection, storage and release according to the Directive 2016/679 of the European Parliament and of the EU Committee on the protection of natural persons with regard to the processing of personal data and on the free movement of such data, and repealing Directive 95/46/EC (General Data Protection Regulation).

## 5. Conclusions

The public UCB banks represent an established source of HSCs for allogeneic transplantation particularly for pediatric patients and patients belonging to HLA minorities. In this report we have presented the policies developed in Greece, a country with high HLA heterogeneity, prioritizing the HLA diversity of UCB banking and optimizing the collection, processing, storage and characterization procedures to improve the economic sustainability of the public UCB banks system. The high rejection rate of the donated UCB units due to the strict acceptance criteria for HSCT purposes, in association with the high application of the haploidentical HSCT, have resulted in a significant limitation of the UCB use in the clinic, with a consequent impact on the economic sustainability of the public UCB banks. The perspective of recycling the inappropriate for HSCT units or using the high-quality excess units for isolation and expansion of immune and stem cell populations for cell engineering/reprogramming processes and for production of cell-derivatives for research and clinical applications, extends the therapeutic value of the UCB and increases the sustainability potential of the UCB banks [18,21,22,23,35,38,39,40,41,42,52,65,68,70,75,78,79,80,81,82,83,84,85,86,97,98,99,100]. Novel applications in adoptive immunotherapy, regenerative medicine and specialized transfusion are anticipated not only to maximize the use of the UCB but also to minimize the financial deficit of the public UCB banks through reimbursement procedures. The communication of these promising UCB clinical applications to the lay public is anticipated also to motivate the families for even more altruistic UCB donations. The collaborative approaches and outcomes of the Greek public UCB banks presented in the current report, for the efficient use of UCB in the field of allogeneic HSCT for populations with high HLA heterogeneity that are underrepresented in international repositories as well as in novel fields of medicine, add value to the international efforts aiming to sustainably expand the public UCB banking system. The expansion of UCB banks from transplantation banks to banks that support cell therapy, regenerative medicine and transfusion medicine will ensure that high quality UCB banks can not only survive but even thrive, offering treatment opportunities for patients with a broad spectrum of hematologic, genetic, and degenerative diseases.

## Figures and Tables

**Table 1:** Criteria used for UCB units selection for unrelated HSCT *.

TEST	Criteria
Total nucleated cell (TNC) count	≥500 × 10^6^
TNC recovery	≥60%
Viable CD34 cell count	≥1.25 × 10^6^
Viability of CD34 cells	≥70% (on thawed representative sample)
Viability of TNC count	≥85%
Potency	growth or positive result of potency (on thawed representative sample)
Microbial screen	negative result for aerobes, anaerobes, fungus
Donor Testing for Infectious disease	negative result for: HIV1, HIV2, Hepatitis B, Hepatitis C, HTLV I, HTLV II, Syphilis, CMV, and other additional tests for infectious transmissible agents in accordance to applicable law, or institutional policy

Abbreviations: UCB, umbilical cord blood; HSCT, hematopoietic stem cell transplantation; HIV, human immunodeficiency virus; HTLV, human T-cell lymphotropic virus; CMV, cytomegalovirus. * For other clinical applications of UCB, the criteria for cell product sterility, donor testing for infectious diseases, effector cell viability and minimum effector cell count for the respective application should be met.

**Table 2:** Representative UCB-based initiatives in the field of HSCT and beyond, in the Greek public banking system.

Field of Research	References
**HSCT**	
HLA immunoprofiling of the Greek population based on the available data of the public UCB banks and bone marrow donor registries and comparison with established international registries; development of a model to increase the efficacy and cost-effectiveness of the public UCB banks based on the population HLA diversity.	[27,28]
**Off-the-shelf adoptive immunotherapy**	
Production of dendritic cells by UCB-derived CD34^+^ cells stimulating the generation of bivalent leukemia-specific T-cells targeting Wilms tumor 1 and preferentially expressed antigen in melanoma antigens.	[35]
Generation of a compact, “all-in-one”, T-cell product of UCB origin, called “LEVIS” (LEukemia-VIrus-specific T-cells), simultaneously targeting four viruses and two common leukemia associated antigens.	[40]
Studies on the isolation, quantification, functional characterization, storage and expansion of the myeloid derived suppressor cells (MDSCs) from UCB units.	[54]
**Regenerative medicine**	
*Mesenchymal stem/stromal cells (MSCs)*	
Studies on the quantitative, functional and differentiation properties of UCB- and Warton Jelly (WJ)-derived MSCs.	[62]
Studies combining MSCs with scaffolds to explore the differentiation potential of UCB- and WJ-derived MSCs.	[63,64]
Studies on methodologies for long-term storage of WJ with maintenance of MSC survival, proliferation, differentiation properties.	[65]
*Induced pluripotent stem cells (iPSCs)*	
Participation in the Cooperation in Science and Technology HAPLO-iPS Action aiming to develop super donor iPSC lines from UCB units homozygous for common HLA European haplotypes.	https://www.cost.eu/actions/CA21151/ (accessed on 15 February 2024)
**Specialized Transfusion Medicine**	
Evaluation of the qualitative properties of platelet lysates obtained from small volume, non-suitable for HSCT UCB units, in terms of growth factor content, wound healing and angiogenesis properties.	[85,86]
Participation in a European network of public UCB banks to generate and promote protocols for the fractionation of inappropriate for HSCT UCB units into (a) UCB plasma rich platelet for the use in skin ulcers, (b) UCB platelet poor plasma for the preparation of eye drops, and (c) leukoreduced red blood cells for possible transfusion of neonates.	[22]
Clinical study using UCB-derived plasma rich platelet for the treatment of diabetic patients with peripheral arterial disease and critical limb ischemia substituting the standard autologous plasma rich platelet treatment.	[87], unpublished data
**Other research initiatives with clinical potential**	
Investigation of the distinct signature characteristics of microparticles derived from UCB CD34^+^ cells and their potential use in the clinics.	[96]

Abbreviations: UCB, umbilical cord blood; HSCT, hematopoietic stem cell transplantation.

## Data Availability

All data presented in the report from research studies performed by the authors are available in the cited references whereas unpublished data can be available upon request from the corresponding author. The Graphical Abstract, Figure 2 and Figure 3 were created with license from BioRender.com.
